# Maternal and perinatal outcomes in pregnant women with BMI >50: An international collaborative study

**DOI:** 10.1371/journal.pone.0211278

**Published:** 2019-02-04

**Authors:** Stephen J. McCall, Zhuoyang Li, Jennifer J. Kurinczuk, Elizabeth Sullivan, Marian Knight

**Affiliations:** 1 National Perinatal Epidemiology Unit (NPEU), Nuffield Department of Population Health, University of Oxford, Oxford, United Kingdom; 2 The Australian Centre for Public and Population Health Research, Faculty of Health, University of Technology Sydney, Sydney, Australia; Mount Sinai Health System, University of Toronto, CANADA

## Abstract

**Objective:**

To examine the association between maternal BMI>50kg/m^2^ during pregnancy and maternal and perinatal outcomes.

**Materials and methods:**

An international cohort study was conducted using data from separate national studies in the UK and Australia. Outcomes of pregnant women with BMI>50 were compared to those of pregnant women with BMI<50. Multivariable logistic regression estimated the association between BMI>50 and perinatal and maternal outcomes.

**Results:**

932 pregnant women with BMI>50 were compared with 1232 pregnant women with BMI<50. Pregnant women with BMI>50 were slightly older, more likely to be multiparous, and have pre-existing comorbidities. There were no maternal deaths, however, extremely obese women had a nine-fold increase in the odds of thrombotic events compared to those with a BMI<50 (uOR: 9.39 (95%CI:1.15–76.43)). After adjustment, a BMI>50 during pregnancy had significantly raised odds of preeclampsia/eclampsia (aOR:4.88(95%CI: 3.11–7.65)), caesarean delivery (aOR: 2.77 (95%CI: 2.31–3.32)), induction of labour (aOR: 2.45(95% CI:2.00–2.99)) post caesarean wound infection (aOR:7.25(95%CI: 3.28–16.07)), macrosomia (aOR: 8.05(95%CI: 4.70–13.78)) compared a BMI<50. Twelve of the infants born to women in the extremely obese cohort died in the early neonatal period or were stillborn.

**Conclusions:**

Pregnant women with BMI>50 have a high risk of inferior maternal and perinatal outcomes.

## Introduction

Obesity, defined as a BMI >30kg/m^2^, has been shown to be associated with a number of poor perinatal and maternal outcomes such as hypertensive disorders, metabolic disorders, iatrogenic intervention and poor perinatal outcomes including macrosomia, prematurity and stillbirth [1, 2]. As obesity in pregnancy affects a substantial proportion of women, it is estimated 16% of pregnant women have a BMI >30 in the UK, the related complications and conditions represent a significant public health problem [3].

There is a growing body of evidence examining super obesity or extreme obesity in pregnancy, defined as a BMI ≥50 kg/m^2^ at any point during pregnancy [4–8]. These studies have shown that pregnant women with BMI≥50 kg/m^2^ compared to pregnant women with lower BMIs are at an increased risk of multiple adverse maternal and perinatal outcomes. Due to the relatively low prevalence of BMI ≥50 in pregnancy these studies have had either been completed over a long period of time or on a national basis to include a sample size sufficient to allow precise estimates of effect. Despite a number of national studies examining pregnant women with BMI≥50 they have been limited in their ability to examine severe but rare maternal and perinatal outcomes such as thrombotic events and perinatal deaths. Meta-analysis of observational studies is often limited by heterogeneity of populations and measures used across studies. The opportunities to combine data from separate studies completed in different countries are infrequent due to differences in data collection methods and incomparability.

The aim of this study was to examine maternal and perinatal outcomes in pregnant women with BMI of >50 from the UK and Australia.

## Materials and methods

### Study population and design

**Fig 1:**
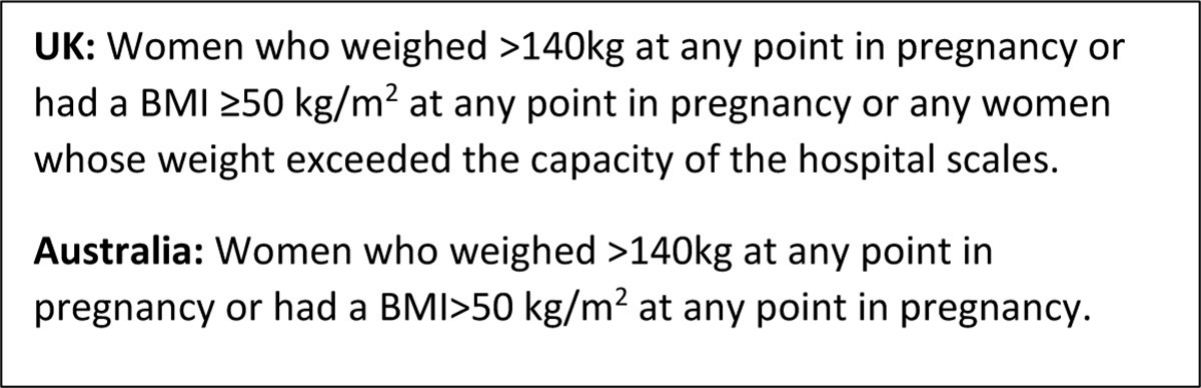
Definitions used to identify the cohorts by country.

This was a collaborative international population-based cohort study using secondary data analysis of two national studies undertaken in Australia and the United Kingdom [6, 8]. The exposed cohort in this study was pregnant women with BMI>50 and the comparison (unexposed) cohort were those with a BMI<50 kg/m^2^. The definitions were harmonised for the purposes of this analysis to include women who had a BMI>50 at any point during pregnancy, see Fig 1.

In each nation, data were collected using national obstetric surveillance systems namely, the United Kingdom Obstetric Surveillance System (UKOSS) and the Australasian Maternity Outcomes Surveillance System (AMOSS). The respective methodologies of each surveillance systems has been explained in detail elsewhere [8–10]. Information about pregnant women with BMI>50 was collected from consultant-led obstetric centres in the UK and from hospitals with over 50 births per year in Australia, using the respective national case definitions (box 1). These are the units in which these women are likely to be delivered.

The UK comparison cohort was identified as the women who delivered in the same unit immediately before the women with BMI>50 but whose BMI was lower. The same questionnaire was used to collect data from both groups of women. Australia’s comparison cohort was identified from data collected in two different AMOSS studies (placenta accreta and peripartum hysterectomy) as the two women delivering immediately prior to the identified cases of placenta accreta or peripartum hysterectomy, and who had a BMI<50 kg/m^2^. In the Australian study, a general questionnaire was used to collect data about both the comparison and extremely obese groups, while the extremely obese group had an additional tailored questionnaire. All data were collected anonymously.

### Data and data collection

Covariates, outcomes and management variables relevant to the research question were identified *a priori* guided by literature review. These desired variables were then included in the analysis if they were available in both datasets. A mapping exercise assessed the comparability of variables between the AMOSS and UKOSS data collection forms. Variables where coding or definitions differed were harmonised if a common definition or coding could be found. Variables where no common definition could be found were excluded from the analysis these included socioeconomic status, gestational diabetes, admission to an intensive therapy unit and postpartum haemorrhage. Ethnicity was not available in the Australian dataset.

Covariates available for analysis were age, smoking status during pregnancy, previous pregnancy problems, pre-existing medical problems, pre-existing hypertension, parity and multiple pregnancy. Management and outcomes collected were those recorded in the hospital records’ of the woman. Management and maternal outcomes assessed in this study were hypertensive disorders during pregnancy, induction of labour, caesarean delivery, post-caesarean wound infection and thrombotic event. Perinatal outcomes explored were perinatal death, stillbirth (>24weeks gestation), preterm birth (<37 weeks), very preterm birth (<32 weeks), birthweight, macrosomia (birthweight ≥4500 grams), congenital abnormality, infant respiratory problem and Apgar score <7 at 5 minutes.

Previous studies using UKOSS data have shown that the distribution of missing data for each variable was not missing at random; as a result multiple imputation was not considered appropriate [11]. For each category of a variable, a proxy category was used to report the missing data for that variable. In a sensitivity analysis, complete case analysis was used to assess the impact of using proxy variables.

The sample size was predetermined by the size of the existing studies; therefore the sample was fixed at 932 pregnant women with BMI>50 and 1232 pregnant women with BMI<50. For the lowest frequency outcome (stillbirth), which had an incidence of 8 per 1000 in the unexposed group, given the sample size the minimum detectable odds ratio with 80% power at the 5% statistical significance level was 3.55 or greater. For the highest frequency outcome, which had an incidence of 26.9% (caesarean delivery) in the unexposed group, the minimum detectable odds ratio with 80% power at the 5% significance level was 1.31 or greater.

### Statistical analysis

Difference in characteristics between those who had a BMI>50 and those with a BMI<50 were assessed using Chi Square tests or Wilcoxon rank sum tests. Each outcome was individually modelled using an unconditional logistic regression model, presented as odds ratios with 95% confidence intervals. To account for clustering of infants within mothers’ with multiple birth, robust estimates of variance were calculated for perinatal outcomes [12]. Collinearity was assessed between all plausible linear associations prior to multivariable analysis, using Pearson’s correlation coefficient.

Only outcomes that were statistically significant at the univariable level were included in the multivariable analysis. A forward stepwise modelling strategy was used to sequentially add potential covariates to the univariable model; results were examined after the addition of each variable. A covariate was included in the final model if it significantly improved the fit of the data as assessed by likelihood ratio tests at the 5% significance level. STATA V.13 was used to complete statistical analysis (STATA CORP, Texas, USA).

In an additional sensitivity analysis, the comparison group was also restricted to pregnant women who had a BMI<30 to assess the impact on the magnitude of identified associations in the multivariable analysis.

### Ethics committee approval

Approval for the obtained secondary use of Australia data obtained was from the Human Research Ethics Committee (HREC) (Ref no. HREC/09/CIPHF/21), New South Wales, Australia. Ethics committee approval for secondary analysis of anonymous data was not required in the UK.

## Results

During the period September 2007-August 2008, 617 pregnant women with BMI>50 kg/m^2^ were identified through the UK Obstetric Surveillance System. Between January—October 2010, 315 pregnant women with BMI>50 kg/m^2^ were identified using the Australasian Maternity Outcomes Surveillance System.

Table 1 presents the characteristics and outcomes of the pooled cohorts of extremely obese women (BMI>50 kg/m^2^) and comparison women (BMI<50 kg/m^2^) from UKOSS and AMOSS. The median BMI was 53 kg/m^2^ (interquartile range (IQR) 51–56) in the extremely obese cohort while the median BMI in the comparison cohort was 25 kg/m^2^ (IQR 22–28). The extremely obese women were on average slightly older (31yrs vs. 30yrs, p <0.001) compared to comparison women. A significantly higher proportion of extremely obese women had antihypertensive medications prior to pregnancy (6.3% vs. 0.5%); multiparity (67.1% vs. 57.9%); a history of previous caesarean deliveries (21.8% vs. 13.5%); and pre-existing diabetes (7.9% vs. 1.2%).

**Table 1:** Sociodemographic characteristics and previous medical problems in pregnant women with BMI>50 and comparison women with BMI<50 during pregnancy.

		Number (%) of obese women (n = 932)		Number (%) of comparison women (n = 1232)		P-value
*Sociodemographic characteristics*						
Age	*Mean (Std)*	31	(5.7)	30	(6.0)	<0.001
BMI at booking	*Median (IQR)*	53	(51–56)	24.5	(21.8–28.4)	<0.001
Smoking status	*Never/ex-smoker*	684	(73.4)	945	(76.7)	
	*Smoked during pregnancy*	230	(24.7)	246	(20)	0.014
	*Missing*	18	(1.9)	41	(3.3)	
*Known previous medical history*						
Known cardiac disease	*None*	921	(98.8)	1,212	(98.4)	
	*Yes*	7	(0.8)	8	(0.6)	0.786
	*Missing*	4	(0.4)	12	(1)	
Known renal disease	*None*	919	(98.6)	1,213	(98.5)	
	*Yes*	9	(1)	7	(0.6)	0.290
	*Missing*	4	(0.4)	12	(1)	
Known mental health issues	*None*	855	(91.7)	1,155	(93.8)	
	*Yes*	73	(7.8)	65	(5.3)	0.017
	*Missing*	4	(0.4)	12	(1)	
Known asthma	*None*	818	(87.8)	1,159	(94.1)	
	*Yes*	110	(11.8)	62	(5)	<0.001
	*Missing*	4	(0.4)	11	(0.9)	
Previous caesarean deliveries	*None*	419	(45)	542	(44)	
	*Yes*	203	(21.8)	166	(13.5)	<0.001
	*Not applicable*	306	(32.8)	519	(42.1)	
	*Missing*	4	(0.4)	5	(0.4)	
Parity	*Nulliparous*	306	(32.8)	519	(42.1)	<0.001
	*Multiparous*	625	(67.1)	713	(57.9)	
	*Missing*	1	(0.1)	0	(0)	
Hypertension prior to pregnancy	*None*	870	(93.3)	1,220	(99)	
	*Yes*	59	(6.3)	6	(0.5)	<0.001
	*Missing*	3	(0.3)	6	(0.5)	
Pre-existing diabetes	*None*	858	(92.1)	1,211	(98.3)	
	*Yes*	74	(7.9)	15	(1.2)	<0.001
	*Missing*	0	(0)	6	(0.5)	
Insulin dependent	*Yes*	21	(2.3)	5	(0.4)	0.062

### Maternal outcomes

Table 2 shows current pregnancy characteristics and maternal outcomes in the extremely obese cohort and the comparison group. Pregnant women with BMI>50 had higher odds of hypertensive disorders during pregnancy, pregnancy induced hypertension and preeclampsia compared to comparison women.

**Table 2:** Current pregnancy details and complications in pregnant women with BMI>50 and comparison women with BMI<50 during pregnancy.

* *		Number (%) of obese women (n = 932)		Number (%) of comparison women (n = 1232)		Unadjusted Odds Ratios	95% Confidence intervals	P-value
Multiple pregnancy	*No*	908	(97.4)	1,211	(98.3)	1		
	*Yes*	24	(2.6)	20	(1.6)	1.60	(0.88–2.92)	0.124
	*Missing*	0	(0)	1	(0.1)			
Hypertensive disorder during pregnancy	*No*	708	(76)	1,167	(94.7)	1		
	*Yes*	216	(23.2)	52	(4.2)	6.85	(4.99–9.40)	<0.001
	*Missing*	8	(0.9)	13	(1.1)			
Pregnancy induced hypertension	*No*	796	(85.4)	1,195	(97)	1		
	*Yes*	128	(13.7)	24	(1.9)	8.01	(5.13–12.50)	<0.001
	*Missing*	8	(0.9)	13	(1.1)			
Preeclampsia and eclampsia	*No*	836	(89.7)	1,191	(96.7)	1		
	*Yes*	88	(9.4)	28	(2.3)	4.48	(2.99–6.91)	<0.001
	*Missing*	8	(0.9)	13	(1.1)			
Induction of labour	*No*	575	(61.7)	955	(77.5)	1		
	*Yes*	343	(36.8)	271	(22)	2.10	(1.74–2.54)	<0.001
	*Missing*	14	(1.5)	6	(0.5)			
Caesarean delivery	*No*	446	(47.9)	900	(73.1)	1		
	*Yes*	473	(50.8)	332	(26.9)	2.87	(2.40–3.44)	<0.001
	*Missing*	13	(1.4)	0	(0)			
Wound infection in those with caesarean delivery	*No*	391	(42)	310	(25.2)	1		
	*Yes*	71	(7.6)	7	(0.6)	8.04	(3.65–17.73)	<0.001
	*N/A*	459	(49.2)	900	(73.1)			
	*Missing*	11	(1.2)	15	(1.2)			
Thrombotic event	*No*	918	(98.5)	1,231	(99.9)	1		
	*Yes*	7	(0.8)	1	(0.1)	9.39	(1.15–76.43)	0.036
	*Missing*	7	(0.8)	0	(0)			

Complete case analysis used for the unadjusted analysis.

Pregnant women with BMI>50 had significantly higher odds of caesarean delivery and induction of labour than the comparison group. Extremely obese women who had a caesarean delivery, had a significantly higher odds of wound infection than the comparison group. Extremely obese women had a nine-fold increase in the odds of thrombotic events compared to those with a BMI<50 during pregnancy.

### Perinatal outcomes

Twelve of the infants born to women in the extremely obese cohort died in the early neonatal period or were stillborn (Table 3). Maternal BMI>50 during pregnancy was associated with raised odds of perinatal death (uOR: 1.78 (95%:0.75–4.25)) and stillbirth (uOR: 1.50 (95%CI: 0.58–3.90)), although these associations were not statistically significant. Although there was a raised odds of preterm birth, very preterm birth and congenital abnormality none of these associations were statistically significant (Table 3).

**Table 3:** Perinatal outcomes in infants born to pregnant women with BMI>50 and to comparison women with BMI<50 during pregnancy.

		Number (%) of infants from obese women		Number (%) of infants from comparison women		Unadjusted Odds Ratios	95% Confidence intervals	P-value
Perinatal death*	*No*	927	(98.5)	1,239	(99.2)	1		
	*Yes*	12	(1.3)	9	(0.8)	1.78	(0.75–4.25)	0.192
	*Missing*	2	(0.2)	1	0.1			
Still birth ≥24weeks gestation	*No*	930	(98.8)	1,239	(99.2)	1		
	*Yes*	9	(1)	8	(0.6)	1.50	(0.58–3.90)	0.407
	*Missing*	2	(0.2)	2	(0.2)			
Preterm birth	*No*	831	(89.2)	1,135	(91.5)	1		
	*Yes*	97	(10.4)	103	(8.3)	1.29	(0.93–1.78)	0.126
	*Missing*	4	(0.4)	3	(0.2)			
Very preterm birth	*No*	915	(98.2)	1,229	(99)	1		
	*Yes*	13	(1.4)	9	(0.7)	1.94	(0.76–4.94)	0.164
	*Missing*	4	(0.4)	3	(0.2)			
Birthweight	*Mean (StD)*	3613.2	(24.1)	3,342	(17.82)	-		
Macrosomia (>4500 grams)	*No*	844	(90.6)	1,221	(98.4)	1		
	*Yes*	86	(9.2)	17	(1.4)	7.32	(4.32–12.40)	<0.001
	*Missing*	2	(0.2)	3	(0.2)			
Congenital anomaly	*No*	904	(97)	1,207	(97.3)			
	*Yes*	16	(1.7)	22	(1.8)	0.97	(0.50–1.88)	0.931
	*Missing*	12	(1.3)	12	(1.0)			
Infant respiratory problem	*No*	906	(97.2)	1,221	(98.4)			
	*Yes*	21	(2.3)	13	(1.0)	2.18	(1.02–4.66)	0.045
	*Missing*	5	(0.5)	7	(0.6)			
5-min Apgar score <7	*No*	883	(94.7)	1,201	(96.8)			
	*Yes*	27	(2.9)	19	(1.5)	1.93	(1.07–3.50)	0.03
	*Missing*	22	(2.4)	21	(1.7)			

*Fetal deaths that have occurred after ≥24 weeks of gestational age and before 7 completed days after birth. Complete case analysis used for the unadjusted analysis.

### Multivariable analysis

#### Maternal outcomes

There were no maternal deaths. Pregnant women with BMI>50 had over a four fold increase in the odds of preeclampsia and eclampsia (adjusted odds ratio (aOR): 4.88 (95%CI: 3.11–7.65)) and nine fold increase in the odds of pregnancy induced hypertension (aOR: 9.09 (95%CI:5.75–14.38) compared to pregnant women with BMI<50, after adjusting for smoking status, pre-existing diabetes and parity (Table 4). Pregnant women with BMI>50 had over two times the odds of having a caesarean delivery (aOR: 2.77 (95%CI: 2.31–3.32)) compared to those with a BMI<50, after adjusting for previous caesarean delivery and previous pregnancy problems (Table 4). Pregnant women with BMI>50 who had a caesarean delivery had seven times the odds of having a wound infection compared to comparison women (aOR: 7.25, 95%CI: 3.28–16.07), after controlling for pre-existing diabetes.

**Table 4:** Adjusted odds ratio (aOR) associated with maternal BMI>50 during pregnancy.

	Model A			Model B			Model C		

*Maternal outcomes*	aOR	95% CI	P-value	aOR	95% CI	P-value	aOR	95% CI	P-value
Preeclampsia & eclampsia									
*BMI<50 kg/m*^*2*^	1			1			1		
*BMI>50 kg/m*^*2*^	4.88	(3.11–7.65)	<0.001	6.53	(3.76–11.34)	<0.001	4.81	(3.06–7.55)	<0.001
Pregnancy induced hypertension									
*BMI<50 kg/m*^*2*^				1			1		
*BMI>50 kg/m*^*2*^	9.09	(5.75–14.38)	<0.001	8.45	(5.07–14.09)	<0.001	8.61	(5.44–13.64)	<0.001
Caesarean delivery									
*BMI<50 kg/m*^*2*^	1			1			1		
*BMI>50 kg/m*^*2*^	2.77	(2.31–3.32)	<0.001	3.34	(2.65–4.21)	<0.001	3.07	(2.49–3.79)	<0.001
Induction of labour									
*BMI<50 kg/m*^*2*^	1			1			1		
*BMI>50 kg/m*^*2*^	2.45	(2.00–2.99)	<0.001	2.64	(2.12–3.29)	<0.001	2.48	(2.02–3.03)	<0.001
Wound infection									
*BMI<50 kg/m*^*2*^	1			1			1		
*BMI>50 kg/m*^*2*^	7.25	(3.28–16.07)	<0.001	11.72	(3.63–37.80)	<0.001	7.25	(3.28–16.07)	<0.001
***Perinatal outcomes***									
Macrosomia*									
*BMI<50 kg/m*^*2*^	1			1			1		
*BMI>50 kg/m*^*2*^	8.05	(4.70–13.78)	<0.001	9.75	(5.06–18.81)	<0.001	8.05	(4.70–13.78)	<0.001
5-min Apgar score <7*									
*BMI<50 kg/m*^*2*^	1			1			1		
*BMI>50 kg/m*^*2*^	2.03	(1.13–3.66)	0.02	2.13	(1.12–4.07)	0.02	2.03	(1.13–3.66)	0.02
Respiratory problem*									
*BMI<50 kg/m*^*2*^	1			1			1		
*BMI>50 kg/m*^*2*^	2.00	(0.91–4.42)	0.09	1.77	(0.76–4.13)	0.18	2.00	(0.91–4.42)	0.09

Model A: proxy variable for missing values. Model B: comparison group BMI <30. Model C: complete case analysis. Pre-eclampsia and pregnancy induced hypertension: adjusted for smoking status, pre-existing diabetes and parity. Caesarean delivery: adjusted for previous caesarean delivery, previous pregnancy problems. Wound infection: adjusted for pre-existing diabetes. Macrosomia: adjusted for gestational age. Apgar < 7 @ 5min: adjusted for gestational age and parity. Respiratory problem: gestational age at delivery.

*Calculated using robust standard errors.

#### Perinatal outcomes

Infants born to pregnant women with BMI>50 were eight times more likely to have macrosomia (aOR: 8.05 (95%CI: 4.70–13.78)) than infants born to women with BMI<50 after adjusting for gestational age at delivery (Table 3). There was a two times (aOR: 2.03 (95% CI: 1.13–3.66)) higher odds of a low Apgar score at 5 minutes among the babies of pregnant women with BMI>50 after adjusting for gestational age at delivery. There was raised odds of an infant respiratory problem/respiratory distress syndrome (aOR: 2.00 (0.91–4.42)), however this was not statistically significant.

The sensitivity analyses shown in models B (which restricted the comparison to women with BMI <30) and C (complete case analysis model) did not materially change the results.

## Discussion

Maternal BMI>50 during pregnancy was associated with an increased likelihood of a number of adverse maternal and perinatal outcomes including hypertensive disorders, macrosomia, a low Apgar score at 5 minutes and caesarean birth. Extreme maternal obesity in both countries was associated with increased odds of potentially preventable outcomes such as thrombotic events and wound infection. This study has shown the combination of two national studies increased the statistical power of testing and precision to estimate the incidence of more common maternal and perinatal outcomes in extremely obese women.

### Strengths and limitations

This study has shown that it is feasible to combine data from two nations examining a rare exposure in pregnancy. Using similar case notification systems and data collection forms. Combining the data increased the sample size and hence the precision of the estimates of effect. The examination of the upper end of the BMI continuum has shown that women with an extremely high BMI have a higher risk of adverse maternal and perinatal outcomes than those with a lower BMI.

The combining of two independent studies from different maternal populations had a number of limitations. Firstly, the definition of extreme obesity was not identical which led to the exclusion of two women with a BMI = 50 from the dataset. In addition, some variables identified from the literature were not available in both datasets or were collected using different definitions, which led to an analysis based on only those variables that existed in both datasets and could be harmonised. As a result, important variables such as socioeconomic status, postpartum haemorrhage and gestational diabetes were not available for analysis. In addition, we did not have access to ethnicity from Australia. Data on outcomes that are not collected uniformly across countries need to be interpreted with caution. Furthermore, the comparison population was collected from consultant led units so these women may have had higher rates of complications than women who deliver in midwife led units. The study comparison group was representative of the general population; however, it may underestimate the risk of outcomes when compared to a group with a normal BMI. Gestational weight gain was not collected, however, if a pregnant women presented with BMI>50 kg/m^2^ at any gestational age she would have been eligible for inclusion in the study.

### Interpretation

There was an increase in the odds ratios for most of the outcomes investigated suggesting that a causal relationship may exist between perinatal mortality and extreme obesity. However, this study did not have the power to exclude the role of chance for a substantial number of outcomes and these tended, unsurprisingly, to be the rarer outcomes. Therefore, future international studies of rare complications of pregnancy in combination with rare outcomes will need to aim to include more countries to increase the size of the study population

A recent review of reviews suggested an association between maternal obesity BMI ≥ 30kg/m^2^ and wound infection after a caesarean delivery [13] which is consistent with our data for pregnant women with BMI>50. UK national guidance recommends that prophylactic antibiotics should be routinely administered to all women who undergo a caesarean delivery [14]. Future research should consider the dose women with BMI>50 receive as there is currently little evidence examining weight appropriate antibiotic dosages [15].

### Hypertensive disorders of pregnancy

There is considerable evidence showing an association between maternal obesity and an increased risk of hypertensive disorders [13, 16]; this relationship has also been shown in pregnant women with BMI ≥50 [7, 17, 18]. With larger numbers this analysis was able to more precisely estimate the magnitude of the association, showing that pregnant women with BMI>50 had over six times the odds of preeclampsia compared to a non-obese population with the 95% CI ranging from a fourfold to a 11 fold increased risk.

### Mode of delivery

The results of this study are consistent with the finding from a review which showed obese women were at increased risk of caesarean delivery [19] and with other studies of women with BMI>50 [7, 17, 18]. Despite an increased risk of regional anaesthetic failure and increased difficulty in intubation at surgical interventions, women with BMI>50 were more likely to be delivered by caesarean section [20]. There are many clinical factors which may lead to the decision to deliver an obese women by caesarean section, some of these include: increased risk of shoulder dystocia, failure to progress, infant distress and previous caesarean deliveries [19]. Added to this, as particularly pertinent in women with BMI>50, is the fear of anaesthetic complications if emergency delivery is required. Appropriate planning of mode of delivery is indicated for pregnant women with BMI>50 as additional resources and equipment may be indicated. As a result, the availability of facilities to ensure safe delivery if surgical problems arise should be factored into the decision surrounding mode of delivery.

### Perinatal outcomes

Meta-analysis of observational data has shown that a high pre-pregnancy body mass index is associated with infant macrosomia [21]. In particular, there is a positive relationship between increasing maternal body mass index and higher infant birthweight. The findings of this study are consistent with both the wider literature on obesity in pregnancy [21] and maternal BMI ≥50 [8, 22]. Similarly, the association between maternal obesity with poor infant condition immediately after birth has been supported by both a wider maternal obesity review [23] and studies of pregnant women with BMI>50 [17].

## Conclusions

The findings showed that pregnant women with BMI>50 have a higher risk of poor maternal outcomes and some perinatal outcomes than those with a lower BMI. Obese women appear to have a higher risk of preventable outcomes such as thrombotic events and wound infection, which highlights that there needs to be a more proactive approach to management. Further guidance to aid prevention amongst this patient group is indicated, including assessment of weight when determining therapeutic drug dosages.
